# Impact of simulated microgravity on the growth and proteomic profile of *Enterobacter cloacae*

**DOI:** 10.1128/spectrum.02446-24

**Published:** 2025-04-24

**Authors:** Jonna Ocampo, Rachel E. White, Mariola J. Ferraro, Kelly C. Rice

**Affiliations:** 1 Department of Microbiology and Cell Science, IFAS, University of Florida, Gainesville, Florida, USA; The Ohio State University College of Dentistry, Columbus, Ohio, USA

**Keywords:** *Enterobacter cloacae*, low-shear modeled microgravity, high aspect ratio vessels, oxygenation, proteomics

## Abstract

Previous studies have shown decreased microbial diversity in astronaut gut microbiomes during spaceflight, raising potential health concerns. *Enterobacter cloacae*, a commensal member of the human gastrointestinal tract microbiota, has the potential to cause opportunistic infections in immunocompromised patients. However, little is known about how *E. cloacae* cells adapt to extreme environments such as microgravity, and whether this bacterium is of medical concern for astronauts during long-term spaceflight missions, as astronaut immune systems are compromised by microgravity. To this end, *E. cloacae* growth and proteomic profiles were obtained from high aspect ratio vessel (HARV) cultures grown in the rotary cell culture system (RCCS) in three orientations: low-shear modeled microgravity (LSMMG), normal gravity (NG; oxygenation membrane on bottom), and inverted normal gravity (INV; oxygenation membrane on top). Both NG and INV controls were included to determine if *E. cloacae* LSMMG and INV HARV cultures overlapped in their growth properties and/or proteomics profiles, as was previously observed for *Staphylococcus aureus*. Our results revealed significant proteomic changes in *E. cloacae* that overlapped in LSMMG and INV conditions compared to NG, suggesting that adaptive responses may be driven by both simulated microgravity and orientation of the oxygenation membrane. These findings offer insight into the molecular mechanisms underlying *E. cloacae*’s adaptation to microgravity and its potential health risks for astronauts. This study also underlines the importance of multiple control orientations in ground-based microgravity simulations using the RCCS, guiding future research on microbial behavior in space conditions.

**IMPORTANCE:**

*Enterobacter cloacae* can transition from a gut commensal to an opportunistic pathogen in immunocompromised hosts and in closed environments such as hospitals. This danger can be exacerbated by the emergence of multidrug-resistant *E. cloacae* strains. Astronauts undergo changes in their immune systems during spaceflight that could predispose them to infection and spend extended time in the International Space Station and other closed environments. Therefore, elucidating the impacts of actual spaceflight and simulated microgravity on the biology of *E. cloacae* and other commensal organisms is vital due to the challenges of antibiotic treatment (such as limited shelf life) during extended spaceflight missions. The findings in this study highlight the importance of using multiple control conditions in ground-based microgravity simulations and lay groundwork for future research into microbial adaptation to space and other extreme environments.

## OBSERVATION

*Enterobacter cloacae*, a commensal member of the human gut microbiota and an opportunistic pathogen (1), poses a potential health risk to astronauts in the confined International Space Station (ISS) environment. As an ESKAPE pathogen (a group of specific antimicrobial-resistant bacteria for which effective therapies are critically needed), *E. cloacae* is increasingly recognized as a concern in hospital-acquired infections (2), and spaceflight can alter the human microbiota (3), which could potentially contribute to health disorders ranging from diarrhea, irritable bowel syndrome, and immune system impairments (4). A related species (*Enterobacter bugandensis*) exhibited increased antibiotic resistance properties and was also isolated from the ISS (5). Despite its importance, *E. cloaca*e’s adaptation to spaceflight remains unexplored.

**Fig 1:**
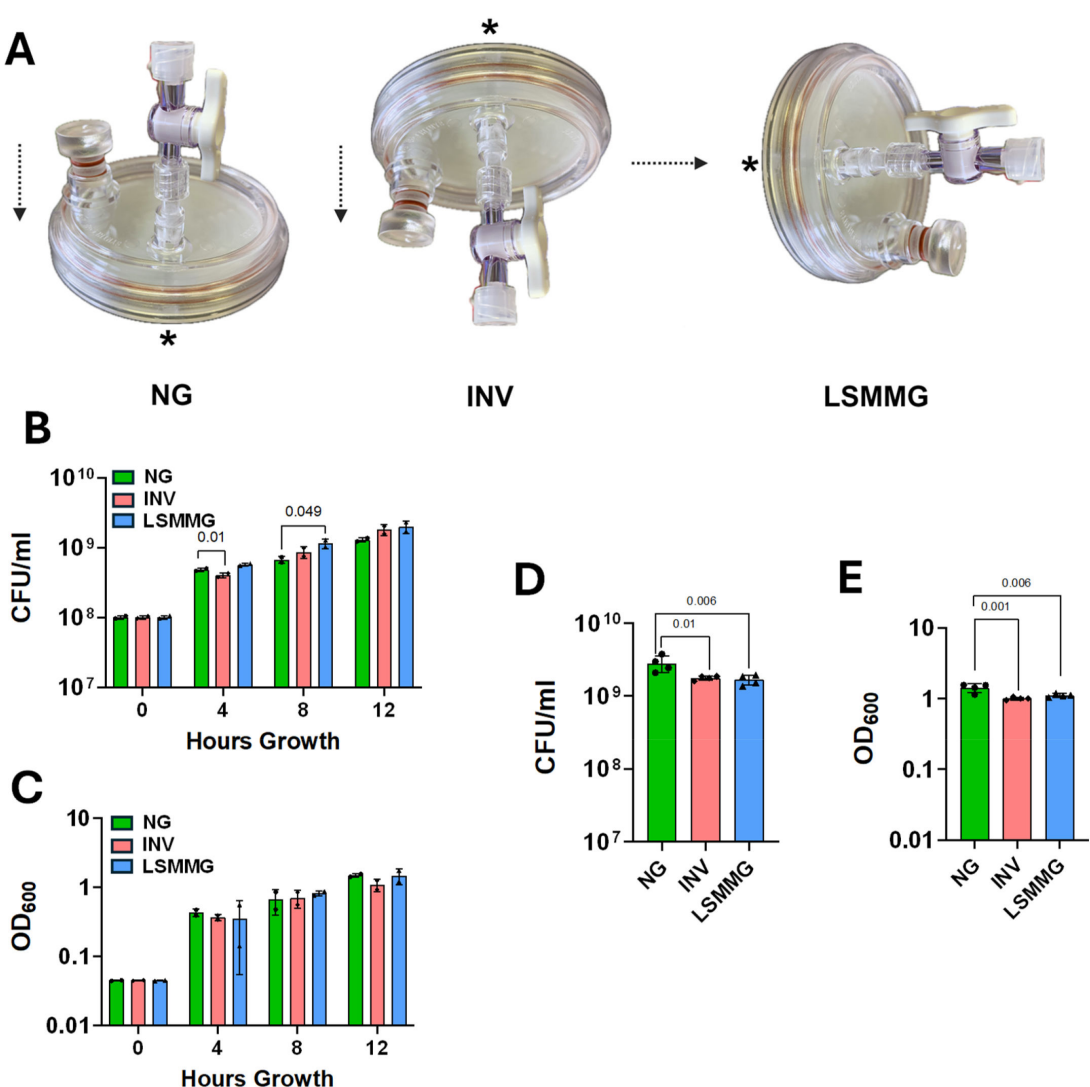
Comparative growth and optical density of *E. cloacae* in HARV cultures under normal gravity, inverted normal gravity, and low-shear modeled microgravity conditions. (**A**) Diagram of LSMMG, NG, and INV HARV orientations. The axis of rotation relative to the gravitational vector is indicated for each orientation by dotted arrows; * indicates the orientation of the HARV oxygenation membrane in each configuration. Created with BioRender.com. (**B and C**) Continual growth of HARV cultures. Fifty milliliter disposable HARVs were filled with lysogeny broth (LB) inoculated with *E. cloacae* ATCC 13047 to an OD_600_ = 0.05. HARV cultures (*n* = 2 independent experiments per growth condition) were grown in NG, INV, and LSMMG orientations for 12 hours at 37°C and 25 RPM. At 4 hour intervals, HARVs were removed from the RCCS, gently shaken to resuspend the bacterial cells, and a small aliquot of each culture was removed and set aside for determining viability counts (**B**) and OD_600_ readings (**C**). This volume was replaced in the HARV with an equal volume of sterile LB, and air bubbles were removed prior to reinitiating HARV rotation. (D and E) Endpoint growth of HARV cultures used for proteomics. Fifty milliliter disposable HARVs were filled with LB inoculated with *E. cloacae* to an OD_600_ = 0.05. HARV cultures (*n* = 4 per growth condition) were grown in LSMMG, NG, and INV orientations for 8 hours at 37°C and 25 RPM, a rotation speed previously used for other bacteria (8). At 8 hours of growth, HARVs were removed from the RCCS, gently shaken to resuspend the bacterial cells, and a 1.5 mL aliquot of each culture was removed and set aside for determining viability counts (**D**) and OD_600_ readings (**E**). The remaining culture was transferred to a 50 mL Falcon tube and centrifuged at 4,500 RPM at 4°C for 10 minutes. Supernatants were removed, and cell pellets were stored at −80°C until lysed and processed for proteomics. For **B–E**, symbols represent sample means, and error bars = standard deviation. Statistical analysis was conducted using ordinary one-way ANOVA followed by Fisher's least significant difference multiple comparisons test; significant (*P* <0.05) differences are indicated.

Ground-based microgravity simulations are an important first step towards understanding *E. cloacae*’s physiology and viability during spaceflight. The rotary cell culture system (RCCS) (6) enables cultivation under low-shear modeled microgravity (LSMMG), whereby high aspect ratio vessels (HARVs) are rotated on an axis perpendicular to the gravitational vector (Fig. 1A). HARV rotation on an axis parallel to the gravitational vector represents the normal gravity (NG) orientation (oxygenation membrane on bottom) (Fig. 1A). In this study, changes in the *E. cloacae* late exponential phase proteome were explored using the RCCS, which included an additional inverted normal gravity (INV) HARV (oxygenation membrane on top; Fig. 1A) control, since *Staphylococcus aureus* LSMMG and INV cultures had demonstrable overlaps in physiological and secreted protein profiles (7).

For proteomics, *E. cloacae* was grown in lysogeny broth (LB; 1% tryptone, 0.5% yeast extract, 0.5% NaCl) at 37°C to late exponential phase (8 hours) in LSMMG, NG, and INV orientations (Fig. 1A) (7). This growth phase was chosen so results could be compared to other studies of late-exponential and/or early-stationary-phase bacterial LSMMG cultures (7–14). We observed that 8 hour LSMMG cultures, which were continually sampled, achieved slightly higher growth yields than NG and INV cultures at this time point (Fig. 1B and C). However, the growth yield of 8 hour endpoint LSMMG and INV HARV cultures (used for proteomics) was modestly reduced compared to NG growth (Fig. 1D and E). The differences in these growth profiles can be attributed to the differences between continual and endpoint sampling. Continual sampling involves removing 1 mL aliquots from each HARV at indicated time points and replacing them with 1 mL sterile media (Fig. 1B and C), whereas endpoint sampling involves growing the HARVs for 8 hours without interruption (Fig. 1D and E). Additionally, the percent reductions in CFU/mL means of INV and LSMMG endpoint cultures were 37% and 41%, respectively, compared to NG culture, whereas percent reductions in OD means were 29% and 22%, respectively (Fig. 1D and E). These data suggest that a small number of dead cells may contribute to the proteomics results.

**Fig 2:**
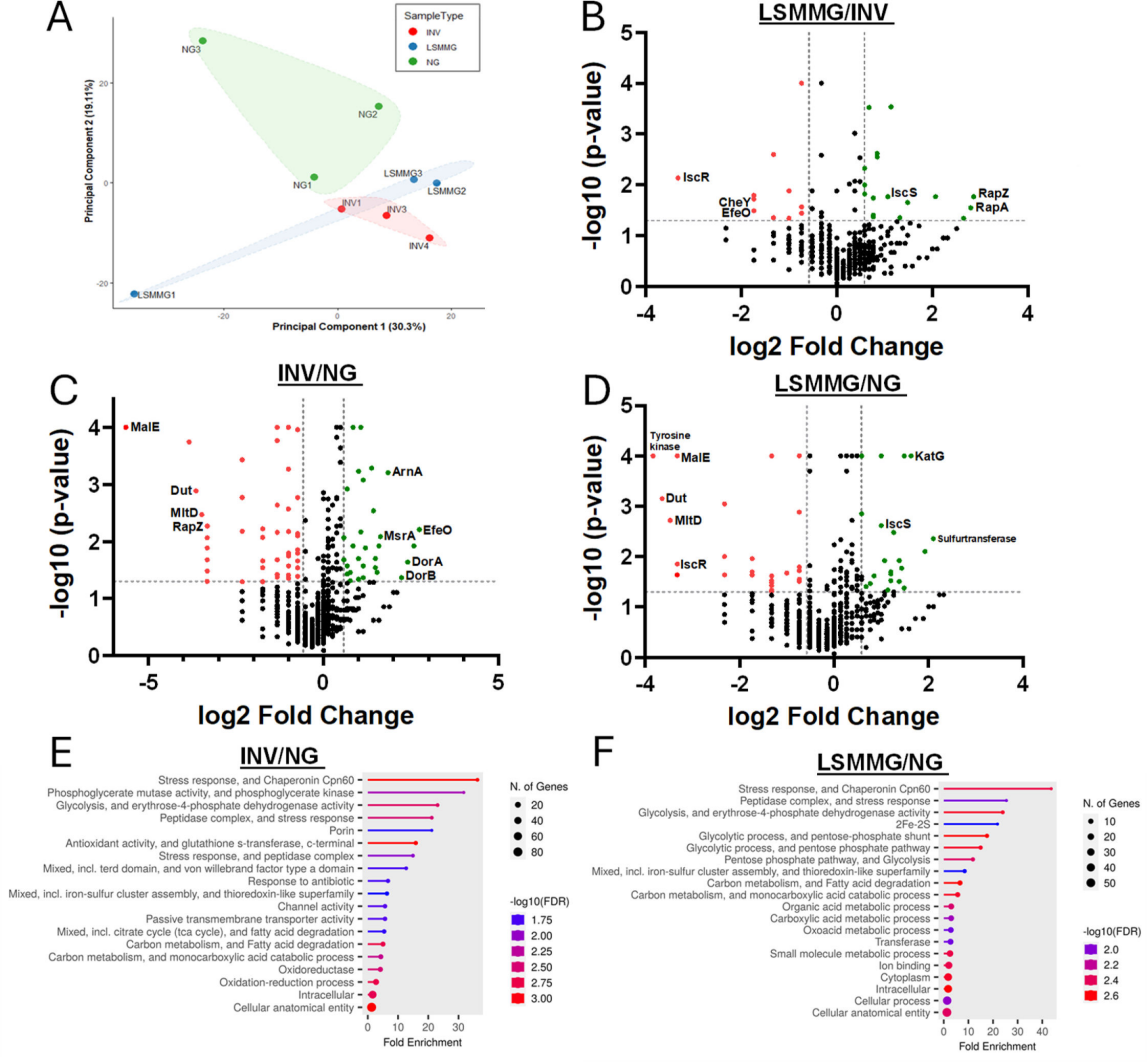
Proteomic analysis of *E. cloacae* responses under NG, INV, and LSMMG conditions. *E. cloacae* was cultured under normal gravity (NG), inverted normal gravity (INV), and low-shear modeled microgravity (LSMMG) conditions for 8 hours. Cellular proteins were then extracted by a 5 minute incubation with B-PER containing EDTA-free protease inhibitors (Thermo Scientific), followed by quantification (15). For proteomics, 25 µg of protein per sample was used, with three biological replicates for each condition. Proteins were separated by SDS-PAGE, followed by in-gel trypsin digestion (16). Peptide samples were analyzed using UHPLC coupled to an Orbitrap Fusion mass spectrometer (16). Tandem mass spectra were processed with Proteome Discoverer 2.1 and analyzed using Mascot against the UniProt *E. cloacae* database (5,412 entries) (17). Protein identifications were validated with *Scaffold* (version 4.11.0), accepting identifications with over 95% probability and using the Protein Prophet algorithm for grouping by shared peptides. The protein FDR was 0.9%, and the peptide FDR was 0.06%. Protein quantification was based on weighted spectral counts, with fold changes calculated. Missing values were imputed, and only proteins present in at least two out of three replicates were reported. Statistical significance was determined using Fisher’s exact test, with a *P* value <0.05 and a fold change of at least 1.5 considered significant, as described previously (15, 16). (**A**) Principal component analysis (PCA) illustrating distinct clustering of proteomic profiles across *E. cloacae* samples. Each point represents an individual sample, with groupings based on protein expression patterns. PCA was performed in *R* using *prcomp*, with visualization in *ggplot2*, and groupings highlighted using the *geom_encircle* function from the *ggalt* package. (**B**) Volcano plot for LSMMG/INV showing upregulated and downregulated differentially abundant proteins, filtered from smallest to largest FC. Red and green dots indicate the significantly downregulated and upregulated proteins, respectively. Proteins were filtered by fold change (FC > ± 1.5, *P* <0.05). (**C**) Volcano plot for INV/NG showing upregulated and downregulated differentially abundant proteins. Red and green dots indicate the significantly downregulated and upregulated proteins, respectively. Proteins were filtered by fold change (FC > ± 1.5, *P* <0.05). (**D**) Volcano plot for LSMMG/NG showing upregulated and downregulated differentially abundant proteins, filtered from smallest to largest FC. Red and green dots indicate the significantly downregulated and upregulated proteins, respectively. Proteins were filtered by fold change (FC > ± 1.5, *P* <0.05). (**E–F**) Gene ontology enrichment analysis of differentially abundant proteins. Functional analysis of differentially abundant proteins was conducted using *ShinyGO* (ver. 0.80) with the *E. cloacae* ATCC 13047 STRING db. A minimum of two proteins per pathway was required, with redundancies removed and an FDR cutoff of 0.05. The top 20 pathways by category are displayed for gene ontology enrichment analysis of differentially abundant proteins for INV/NG (**E**) and LSMMG/NG (**F**).

Proteomics was performed on 8 hour endpoint HARV cultures as described in reference (15), identifying 172 statistically significant differentially abundant proteins across the three experimental groups: LSMMG/NG (54 proteins; Table S1), INV/NG (86 proteins; Table S2), and LSMMG/INV (32 proteins; Table S3). All proteomics data are available through NASA’s Open Science Data Repository (OSD-788; https://osdr.nasa.gov/bio/repo/data/studies/OSD-788). Principal component analysis (PCA, Fig. 2A) revealed distinct clustering of LSMMG, NG, and INV samples, with LSMMG samples showing the greatest variability. Hierarchical clustering (Fig. S1A) further supported these group distinctions. Volcano plots (Fig. 2B through D) visualized significantly altered protein abundances in all three comparisons. LSMMG/NG and INV/NG displayed similar patterns of protein upregulation and downregulation (Fig. 2C and D), with a 19.8% overlap between LSMMG/NG and INV/NG, encompassing 26 proteins (Table S4; Fig. S1B). In contrast, smaller overlaps were observed between LSMMG/INV and LSMMG/NG (five shared proteins), and LSMMG/INV and INV/NG (10 shared proteins) (Tables S5 and S6). The substantial overlap between LSMMG/NG and INV/NG, combined with decreased growth of INV and LSMMG endpoint cultures relative to NG (Fig. 1D and E), suggests that LSMMG and INV samples exhibit a subset of shared cellular responses that are distinct from NG.

ShinyGO analysis detected many significantly enriched (FDR < 0.05) gene ontology (GO) terms in the LSMMG/NG (80) and INV/NG (33) comparisons (Fig. 2E and F; Table S7). In contrast, only five significant enrichments were detected in the LSMMG/INV comparison (Table S7). A large overlap in enriched GO terms was observed between LSMMG/NG and INV/NG, specifically in categories related to stress responses, iron-sulfur cluster assembly, glycolysis, and fatty acid degradation (Fig. 2E and F). Overlapped proteins related to these GO terms included downregulation of chaperone proteins GroEL, GroES, and DnaK (18), downregulation of 3-ketoacyl-CoA thiolase (FadA), upregulation of catalase (KatG) (19), and sulfurtransferase proteins (Table S4).

*E. cloacae* INV and LSMMG endpoint cultures (Fig. 1D and E) displayed modestly decreased growth compared to NG cultures. This pattern was also observed in *S. aureus* cultures grown under LSMMG and INV conditions, whereby decreased OD_600_ and CFU measurements were observed starting at mid-exponential growth phase and persisting through stationary phase (7). Although INV and LSMMG *S. aureus* cultures demonstrated metabolic, quorum sensing activity, and secretomic profiles suggestive of low-oxygen physiology (7), this did not appear to be the case for *E. cloacae*, as GO term enrichments related to low-oxygen physiology were notably absent in both LSMMG/NG and INV/NG comparisons (Fig. 2E and F). Instead, *E. cloacae* INV/NG and/or LSMMG/NG comparisons contained several upregulated proteins involved in oxidative stress resistance, including KatG (19), dimethyl sulfoxide reductase subunits A and B (20), methionine sulfoxide reductase (21), and cysteine desulfurase IscS (22). Although we did not test oxidative stress resistance, these proteomic results suggest that *E. cloacae* responds to oxidative stress during LSMMG and INV growth. Both *Salmonella* and *Pseudomonas aeruginosa* late-stationary-phase LSMMG cultures exhibited increased resistance to H_2_O_2_ (9, 12), whereas *Salmonella*, *E. cloacae*, and other *Enterobacteriaceae* LSMMG cultures displayed increased H_2_O_2_ sensitivity when tested at earlier growth phases (14, 23). *Streptococcus mutans* stationary phase LSMMG cells were also more sensitive to oxidative stress (11). Collectively, these data suggest that bacterial susceptibility to oxidative stress in LSMMG is both species and growth phase specific.

This *E. cloacae* study, as well as our recent *S. aureus* LSMMG research (7), demonstrates the importance of including both INV and NG controls in RCCS studies. The INV control accounts for the contribution of the gas permeable membrane orientation to 1 g HARV cultures. However, potential differences in O_2_ availability between NG and LSMMG cultures are biologically relevant to spaceflight (microgravity), as oxygen availability is greatly decreased in spaceflight and the microgravity environment, due to decreases in both convective and diffusive oxygen transport pathways (24, 25). Although oxygenation is maintained in HARVs through the gas-permeable membrane, differences in mixing between LSMMG and NG orientations (9) and/or cell sedimentation or biofilm formation on the oxygenation membrane (7, 9) may occur. *P. aeruginosa* LSMMG cultures had decreased oxygen transfer coefficients relative to NG, although overall differences in bulk oxygen content were not observed (9). The 17 unique LSMMG/INV *E. cloacae* proteins listed in Table S8 may therefore represent the proteome response to LSMMG independent of oxygenation membrane orientation, including upregulated nucleoside diphosphate kinase regulator and LysM domain-containing proteins, and downregulated CheY chemotaxis and TorD family chaperone proteins. Overall, this study reinforces the importance of both NG and INV controls in RCCS studies, while recognizing that environmental factors in addition to oxygenation (nutrient availability, metabolite concentration, sedimentation) may also influence protein expression, turnover, and stability in NG, INV, and LSMMG orientations.

## SUPPLEMENTAL MATERIAL

Supplemental material spectrum.02446-24-s0001.pdf Fig. S1; Tables S1 to S8.

ASM does not own the copyrights to Supplemental Material that may be linked to, or accessed through, an article. The authors have granted ASM a non-exclusive, world-wide license to publish the Supplemental Material files. Please contact the corresponding author directly for reuse.
