# Supplementary material for: Impact of simulated microgravity on the growth and proteomic profile of Enterobacter cloacae
Source: Microbiol Spectr. 2025 Apr 24;13(6):e02446-24. doi: 10.1128/spectrum.02446-24 (PMC12131722; doi:10.1128/spectrum.02446-24)
Supplement: Supplemental material — Fig. S1; Tables S1 to S8. [file spectrum.02446-24-s0001.pdf]

Supplementary Figure

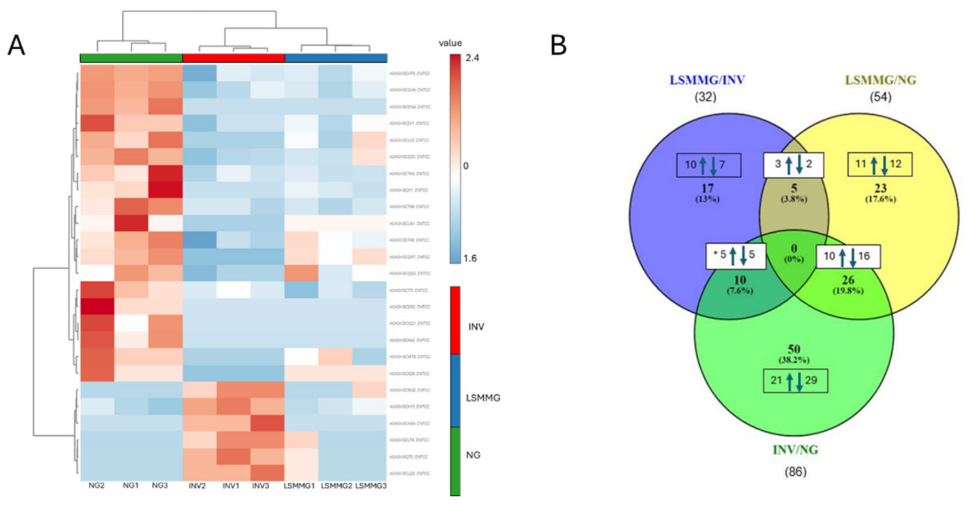

Figure S1. Proteomic analysis of *E. cloacae* responses under NG, INV, and LSMMG conditions. (A) Hierarchical clustering heatmap of the top 25 differentially abundant proteins. The heatmap, generated using MetaboAnalyst 6.0 with autoscaling, clusters proteins and samples based on Euclidean distance and Ward's method (1, 2). The color gradient represents scaled protein abundance. **(B)** Venn diagram of 172 differentially abundant HARV proteins. The diagram shows significant differential abundance among LSMMG/INV (32), LSMMG/NG (54), and INV/NG (86) after 8 hours of HARV rotation. Statistical significance was determined using Fisher's test ($p < 0.05$), with an FDR cutoff of 0.05 and FC thresholds of ≥1.5 and ≤0.60. Analysis was performed using *Venny* 2.1 (3).

Supplementary Table legends

Table S1. List of 54 differentially abundant proteins identified in the LSMMG/NG comparison. The table includes protein names, UniProt accession numbers, fold-change values, and p-values. Proteins with fold changes greater than 1.5 or less than -1.5 and p-values < 0.05 are considered significant.

Table S2. List of 86 differentially abundant proteins identified in the INV/NG comparison. The table includes protein names, UniProt accession numbers, fold-change values, and p-values. Proteins with fold changes greater than 1.5 or less than -1.5 and p-values < 0.05 are considered significant.

Table S3. List of 32 differentially abundant proteins identified in the LSMMG/INV comparison. The table includes protein names, UniProt accession numbers, fold-change values, and p-values. Proteins with fold changes greater than 1.5 or less than -1.5 and p-values

< 0.05 are considered significant.

Table S4. Overlapping proteins identified between the LSMMG/INV and LSMMG/NG comparisons. The table includes five proteins with shared fold-change directionality in both comparisons.

Table S5. Overlapping proteins identified between the LSMMG/NG and INV/NG comparisons. The table lists 26 proteins with consistent fold-change patterns across both comparisons.

Table S6. Overlapping proteins identified between the LSMMG/INV and INV/NG comparisons. The table highlights 10 proteins showing inverse fold-change relationships between the two comparisons.

Table S7. ShinyGO functional enrichment results for LSMMG/NG, INV/NG, and LSMMG/INV comparisons. Functional analysis of differentially abundant proteins was conducted using *ShinyGO* (ver. 0.80) with the *E. cloacae* ATCC 13047 STRING db. A minimum of two proteins per pathway was required, with redundancies removed and an FDR cutoff of 0.05. The table lists all functional enrichments with FDR < 0.05.

Table S8. Unique proteins identified in the LSMMG/INV comparison. The table lists 17 proteins exclusively differentially abundant in the LSMMG/INV comparison.

References cited:

1. Pang Z, Lu Y, Zhou G, Hui F, Xu L, Viau C, Spigelman AF, MacDonald PE, Wishart DS, Li S, Xia J. 2024. MetaboAnalyst 6.0: towards a unified platform for metabolomics data processing, analysis and interpretation. Nucleic Acids Res 52:W398-W406.
2. Pang Z, Zhou G, Ewald J, Chang L, Hacariz O, Basu N, Xia J. 2022. Using MetaboAnalyst 5.0 for LC-HRMS spectra processing, multi-omics integration and covariate adjustment of global metabolomics data. Nat Protoc 17:1735-1761.
3. Oliveros JC. 2007. VENNY. An interactive tool for comparing lists with Venn Diagrams. http://bioinfogp cnb csic es/tools/venny/index html.

Table S1. List of 54 differentially abundant proteins identified in the LSMMG/NG comparison. The table includes protein names, UniProt accession numbers, fold-change values, and p-values. Proteins with fold changes greater than 1.5 or less than -1.5 and p-values < 0.05 are considered significant.

Protein name	Accession Number	Fisher's Exact Test (p < 0.05)	FC +/-
Tyrosine kinase	A0A0H3CQW1_ENTCC	0.0001	-14.29
Deoxyuridine 5'-triphosphate nucleotidohydrolase	A0A0H3CDN4_ENTCC	0.00069	-12.50
Mannitol-1-phosphate 5-dehydrogenase	A0A0H3CGQ1_ENTCC	0.0019	-11.11
Maltodextrin-binding protein	A0A0H3CJY1_ENTCC	0.0001	-10.00
HTH-type transcriptional regulator IscR	A0A0H3CP88_ENTCC	0.014	-10.00
Glutathione S-transferase domain-containing protein	A0A0H3CKK2_ENTCC	0.023	-10.00
Putative hydrolase-oxidase	A0A0H3CN26_ENTCC	0.023	-10.00
Core-binding (CB) domain-containing protein	A0A0H3CH80_ENTCC	0.00086	-5.00
D-isomer specific 2-hydroxyacid dehydrogenase, NAD-binding protein	A0A0H3CMB8_ENTCC	0.0099	-5.00
Ribulose-phosphate 3-epimerase	A0A0H3CUE2_ENTCC	0.023	-5.00
Uridine phosphorylase	A0A0H3CR68_ENTCC	0.011	-3.33
Phosphoenolpyruvate carboxylase	A0A0H3CTI5_ENTCC	0.02	-3.33
Molybdate ABC transporter periplasmic protein	A0A0H3CKX6_ENTCC	0.023	-3.33
Triosephosphate isomerase	A0A0H3CTK6_ENTCC	0.0001	-2.50
3-ketoacyl-CoA thiolase FadA	A0A0H3CT88_ENTCC	0.024	-2.50
DUF853 domain-containing protein	A0A0H3CSD9_ENTCC	0.03	-2.50
Tryptophan--tRNA ligase	A0A0H3CSP8_ENTCC	0.033	-2.50
UPF0265 protein ECL_03331	A0A0H3CND8_ENTCC	0.037	-2.50
3'(2'),5'-bisphosphate nucleotidase CysQ	A0A0H3CE96_ENTCC	0.046	-2.50
Purine nucleoside phosphorylase DeoD-type	A0A0H3CGE5_ENTCC	0.049	-2.50
Outer membrane protein W	A0A0H3CHT9_ENTCC	0.021	-2.00
Co-chaperonin GroES	A0A0H3CG35_ENTCC	0.0001	-1.67
Chaperonin GroEL	A0A0H3CHT6_ENTCC	0.0001	-1.67
Chaperone protein DnaK	A0A0H3CGH6_ENTCC	0.0001	-1.67
Alkyl hydroperoxide reductase C	A0A0H3CHF3_ENTCC	0.0013	-1.67
Pyruvate kinase	A0A0H3CGL4_ENTCC	0.016	-1.67
Succinate dehydrogenase flavoprotein subunit	A0A0H3CL07_ENTCC	0.019	-1.67
AcrA protein	A0A0H3CGL3_ENTCC	0.028	-1.67
Pyrroline-5-carboxylate reductase	A0A0H3CGD3_ENTCC	0.029	-1.67
Transcriptional regulator HU subunit alpha	A0A0H3CDC6_ENTCC	0.031	-1.67
Ornithine carbamoyltransferase	A0A0H3CED3_ENTCC	0.0001	+1.50
3-oxoacyl-(Acyl carrier protein) synthase I	A0A0H3CPT3_ENTCC	0.0014	+1.50
Acetate kinase	A0A0H3CNM6_ENTCC	0.025	+1.50
Na(+)-translocating NADH-quinone reductase subunit A	A0A0H3CJ88_ENTCC	0.028	+1.50
Flagellar hook-associated protein 2	A0A0H3CIC9_ENTCC	0.039	+1.60
2,3-bisphosphoglycerate-independent phosphoglycerate mutase	A0A0H3CEN0_ENTCC	0.024	+1.80
Glycine cleavage system H protein	A0A0H3CQ70_ENTCC	0.0001	+2.00
Cysteine desulfurase IscS	A0A0H3CNC4_ENTCC	0.0024	+2.00
DNA topoisomerase 1	A0A0H3CJ11_ENTCC	0.012	+2.10
50S ribosomal subunit assembly factor BipA	A0A0H3CSQ7_ENTCC	0.046	+2.20

Acetylornithine/succinyldiaminopimelate aminotransferase	A0A0H3CSN4_ENTCC	0.02	+2.30
Protein phosphatase CheZ	A0A0H3CIJ4_ENTCC	0.022	+2.30
Lipoyl synthase	A0A0H3CN60_ENTCC	0.03	+2.30
50S ribosomal protein L32	A0A0H3CKF0_ENTCC	0.0033	+2.40
tRNA uridine(34) hydroxylase	A0A0H3CLS8_ENTCC	0.05	+2.40
Bactoprenol glucosyl transferase	A0A0H3CMZ4_ENTCC	0.012	+2.60
RNA polymerase-associated protein RapA	A0A0H3CEW2_ENTCC	0.031	+2.60
Bifunctional polymyxin resistance protein ArnA	A0A0H3CSZ9_ENTCC	0.017	+2.70
Putative fimbrial protein	A0A0H3CDJ5_ENTCC	0.0001	+2.80
Mannonate dehydratase	A0A0H3CN80_ENTCC	0.042	+2.80
Catalase-peroxidase KatG	A0A0H3CTH7_ENTCC	0.0001	+3.10
Na(+)-translocating NADH-quinone reductase subunit F	A0A0H3CJ92_ENTCC	0.0079	+3.80
Sulfurtransferase	A0A0H3CNB4_ENTCC	0.0044	+4.30

Table S2. List of 86 differentially abundant proteins identified in the INV/NG comparison. The table includes protein names, UniProt accession numbers, fold-change values, and p-values. Proteins with fold changes greater than 1.5 or less than -1.5 and p-values < 0.05 are considered significant.

**Protein name**	**Accession Number**	**Fisher's Exact Test (p < 0.05)**	**FC +/-**
Maltodextrin-binding protein malE	A0A0H3CJY1_ENTCC	0.0001	-50.00
Deoxyuridine 5'-triphosphate nucleotidohydrolase dut	A0A0H3CDN4_ENTCC	0.0013	-12.50
Mannitol-1-phosphate 5-dehydrogenase mtlD	A0A0H3CGQ1_ENTCC	0.0034	-11.11
RNase adapter protein RapZ	A0A0H3CQ97_ENTCC	0.0054	-10.00
3-phosphoshikimate 1-carboxyvinyltransferase aroA	A0A0H3CKY0_ENTCC	0.0086	-10.00
Methyltranfer_dom domain-containing protein ECL_02089	A0A0H3CG83_ENTCC	0.013	-10.00
UMP phosphatase nagD	A0A0H3CLV2_ENTCC	0.021	-10.00
Plasmid-partitioning protein SopB	A0A0H3CU14_ENTCC	0.033	-10.00
Cytidine deaminase	A0A0H3CM78_ENTCC	0.033	-10.00
Tellurite resistance protein TerB	A0A0H3CTJ3_ENTCC	0.033	-10.00
Glutathione S-transferase domain-containing protein ECL_028811	A0A0H3CKK2_ENTCC	0.033	-10.00
Betaine aldehyde dehydrogenase betB	A0A0H3CM26_ENTCC	0.05	-10.00
D-isomer specific 2-hydroxyacid dehydrogenase, NAD-binding protein	A0A0H3CMB8_ENTCC	0.0066	-5.00
Malate synthase	A0A0H3CDD1_ENTCC	0.0066	-5.00
Thiol:disulfide interchange protein	A0A0H3CV23_ENTCC	0.0017	-5.00
Core-binding (CB) domain-containing protein	A0A0H3CH80_ENTCC	0.00037	-5.00
Aldose 1-epimerase	A0A0H3CMI2_ENTCC	0.006	-3.33
NADH-quinone oxidoreductase subunit F	A0A0H3CRL1_ENTCC	0.0085	-3.33
Enolase eno	A0A0H3CEM8_ENTCC	0.022	-3.33
DUF3251 domain-containing protein	A0A0H3CHG2_ENTCC	0.029	-3.33
Sugarporin_N domain-containing protein	A0A0H3CJZ5_ENTCC	0.042	-2.50
3-ketoacyl-CoA thiolase fadA	A0A0H3CT88_ENTCC	0.042	-2.50
Chaperone protein DnaJ	A0A0H3CGV5_ENTCC	0.021	-2.50
ATP-dependent RNA helicase SrmB	A0A0H3CNG0_ENTCC	0.038	-2.50
Pyrroline-5-carboxylate reductase proC	A0A0H3CGD3_ENTCC	0.0023	-2.50
Thioredoxin reductase	A0A0H3CM97_ENTCC	0.0069	-2.50
Arginine transporter, periplasmic binding protein	A0A0H3CP70_ENTCC	0.00017	-2.50
Triosephosphate isomerase tpiA	A0A0H3CTK6_ENTCC	0.0001	-2.50
Tellurium resistance protein TerZ	A0A0H3CW80_ENTCC	0.027	-2.00
Outer membrane protein W	A0A0H3CHT9_ENTCC	0.044	-2.00
Glutathionine S-transferase	A0A0H3CJP8_ENTCC	0.018	-2.00
Universal stress protein	A0A0H3CS33_ENTCC	0.039	-2.00
Transcriptional regulator HU subunit alpha	A0A0H3CDC6_ENTCC	0.016	-2.00
Thiol:disulfide interchange protein	A0A0H3CTR0_ENTCC	0.0067	-2.00
Periplasmic serine endoprotease DegP-like	A0A0H3CF98_ENTCC	0.0027	-2.00
Alkyl hydroperoxide reductase C	A0A0H3CHF3_ENTCC	0.00054	-2.00
Succinate--CoA ligase [ADP-forming] subunit alpha sucD	A0A0H3CPV5_ENTCC	0.0001	-2.00
Co-chaperonin GroES	A0A0H3CG35_ENTCC	0.0001	-2.00
Chaperone protein DnaK	A0A0H3CGH6_ENTCC	0.0001	-2.00
ATP synthase subunit beta atpD	A0A0H3CRS4_ENTCC	0.0001	-2.00

Chaperonin GroEL	A0A0H3CHT6_ENTCC	0.0001	-2.00
AcrA protein	A0A0H3CGL3_ENTCC	0.016	-1.67
OmpF porin	A0A0H3CK46_ENTCC	0.022	-1.67
ATP synthase gamma chain atpG	A0A0H3CVH7_ENTCC	0.0073	-1.67
Peptidoglycan-associated protein pal	A0A0H3CKZ0_ENTCC	0.041	-1.67
Citrate synthase	A0A0H3CN21_ENTCC	0.0079	-1.67
Glutamate/aspartate transport system substrate-binding protein gltI	A0A0H3CPZ7_ENTCC	0.029	-1.67
Tellurium resistance protein TerE	A0A0H3CW88_ENTCC	0.0017	-1.67
Outer membrane porin C ompC	OMPC_ENTCC	0.014	-1.67
Glyceraldehyde-3-phosphate dehydrogenase	A0A0H3CN81_ENTCC	0.00011	-1.67
DNA protection during starvation protein dps	A0A0H3CKP0_ENTCC	0.021	+1.50
L-serine dehydratase	A0A0H3CPY1_ENTCC	0.0086	+1.50
Nucleoid-associated protein ECL_03507	A0A0H3CPF2_ENTCC	0.037	+1.60
Efflux pump membrane transporter acrB	A0A0H3CFX2_ENTCC	0.027	+1.60
Serine-type D-Ala-D-Ala carboxypeptidase	A0A0H3CQ15_ENTCC	0.0012	+1.60
Glucans biosynthesis protein D mdoD	A0A0H3CKA7_ENTCC	0.049	+1.70
50S ribosomal protein L32 rpmF	A0A0H3CKF0_ENTCC	0.035	+1.70
Phosphoribosylaminoimidazole-succinocarboxamide synthase purC	A0A0H3CN29_ENTCC	0.049	+1.70
Fumarate hydratase class I	A0A0H3CLM8_ENTCC	0.012	+1.80
Flagellin fliC	A0A0H3CK20_ENTCC	0.0001	+1.80
Bactoprenol glucosyl transferase	A0A0H3CMZ4_ENTCC	0.046	+2.00
Glyoxylate/hydroxypyruvate reductase A ghrA	A0A0H3CJU6_ENTCC	0.046	+2.00
Acetyltransferase	A0A0H3CHM2_ENTCC	0.02	+2.00
Catalase-peroxidase katG	A0A0H3CTH7_ENTCC	0.00059	+2.00
YcaO domain-containing protein	A0A0H3CP03_ENTCC	0.0068	+2.10
Putative fimbrial protein	A0A0H3CDJ5_ENTCC	0.0001	+2.10
30S ribosomal protein S19	A0A0H3CQI0_ENTCC	0.027	+2.20
Putative tellurium resistance protein TerY	A0A0H3CW66_ENTCC	0.043	+2.20
2,3-bisphosphoglycerate-independent phosphoglycerate mutase gpmI	A0A0H3CEN0_ENTCC	0.00084	+2.20
Protein phosphatase CheZ	A0A0H3CIJ4_ENTCC	0.013	+2.30
DNA topoisomerase 1 topA	A0A0H3CJ11_ENTCC	0.00052	+2.60
Histidine ammonia-lyase hutH	A0A0H3CKW9_ENTCC	0.0029	+2.70
Beta-barrel assembly-enhancing protease bepA	A0A0H3CNZ5_ENTCC	0.02	+2.80
Sulfurtransferase	A0A0H3CNB4_ENTCC	0.029	+2.80
Ornithine decarboxylase	A0A0H3CLT7_ENTCC	0.035	+2.90
Psb2	A0A0H3CQU2_ENTCC	0.012	+3.00
Peptide methionine sulfoxide reductase MsrA	A0A0H3CEZ5_ENTCC	0.0082	+3.10
Bifunctional polymyxin resistance protein ArnA	A0A0H3CSZ9_ENTCC	0.00062	+3.60
Anaerobic dimethyl sulfoxide reductase, B subunit	A0A0H3CKZ2_ENTCC	0.043	+4.70
Carboxypeptidase family protein	A0A0H3CN94_ENTCC	0.043	+4.70
UPF0304 protein ECL_03638	A0A0H3CRL9_ENTCC	0.043	+4.70
DUF2339 domain-containing protein	A0A0H3CRG8_ENTCC	0.023	+5.30
Anaerobic dimethyl sulfoxide reductase, A subunit	A0A0H3CLT8_ENTCC	0.023	+5.30
PTS system, mannose-specific IID component	A0A0H3CHC9_ENTCC	0.012	+6.00
Iron uptake system component EfeO	A0A0H3CLG5_ENTCC	0.0062	+6.70
YncE family protein	A0A0H3CJT0_ENTCC	0.0062	+6.70

Table S3. List of 32 differentially abundant proteins identified in the LSMMG/INV comparison. The table includes protein names, UniProt accession numbers, fold-change values, and p-values. Proteins with fold changes greater than 1.5 or less than -1.5 and p-values < 0.05 are considered significant.

Protein name	Accession Number	Fisher's Exact Test ($p < 0.05$)	FC +/-
HTH-type transcriptional regulator IscR	A0A0H3CP88_ENTCC	0.0073	-10.00
30S ribosomal protein S19	A0A0H3CQI0_ENTCC	0.016	-3.33
Ribulose-phosphate 3-epimerase	A0A0H3CUE2_ENTCC	0.019	-3.33
Chemotaxis regulatory protein CheY	A0A0H3CI56_ENTCC	0.019	-3.33
Cytoplasmic chaperone TorD family protein	A0A0H3CLU7_ENTCC	0.032	-3.33
Iron uptake system component EfeO	A0A0H3CLG5_ENTCC	0.032	-3.33
YncE family protein	A0A0H3CJT0_ENTCC	0.032	-3.33
Putative oxidoreductase	A0A0H3CIC3_ENTCC	0.0025	-2.50
1,4-dihydroxy-2-naphthoyl-CoA synthase	A0A0H3CPN2_ENTCC	0.044	-2.50
DNA protection during starvation protein Dps	A0A0H3CKP0_ENTCC	0.013	-2.00
3-oxoacyl-[acyl-carrier-protein] synthase 3	A0A0H3CNF2_ENTCC	0.045	-2.00
Flagellin	A0A0H3CK20_ENTCC	0.0001	-1.67
Protein RecA	A0A0H3CSP0_ENTCC	0.027	-1.67
Fructose-bisphosphate aldolase	A0A0H3CMX1_ENTCC	0.036	-1.67
Bifunctional protein PutA	A0A0H3CLH0_ENTCC	0.0047	+1.50
Glucose-specific PTS system component	A0A0H3CPZ1_ENTCC	0.01	+1.50
Periplasmic serine endoprotease DegP-like	A0A0H3CF98_ENTCC	0.015	+1.50
Succinate--CoA ligase [ADP-forming] subunit alpha	A0A0H3CPV5_ENTCC	0.0003	+1.60
Arginine transporter, periplasmic binding protein	A0A0H3CP70_ENTCC	0.018	+1.70
UspA domain-containing protein	A0A0H3CQ31_ENTCC	0.039	+1.70
D-amino acid dehydrogenase	A0A0H3CIV3_ENTCC	0.042	+1.70
Protein-export protein SecB	A0A0H3CD34_ENTCC	0.0024	+1.80
Argininosuccinate synthase	A0A0H3CSA7_ENTCC	0.0028	+1.80
Cysteine desulfurase IscS	A0A0H3CNC4_ENTCC	0.017	+2.10
Glycine cleavage system H protein	A0A0H3CQ70_ENTCC	0.00029	+2.20
Aldose 1-epimerase	A0A0H3CMI2_ENTCC	0.044	+2.50
Signal recognition particle protein	A0A0H3CSE5_ENTCC	0.022	+2.80
Methylmalonate-semialdehyde dehydrogenase	A0A0H3CN53_ENTCC	0.017	+4.20
LysM domain-containing protein	A0A0H3CKM3_ENTCC	0.045	+6.30
RNA polymerase-associated protein RapA	A0A0H3CEW2_ENTCC	0.028	+7.00
Regulator of nucleoside diphosphate kinase	A0A0H3CMR3_ENTCC	0.028	+7.00
RNase adapter protein RapZ	A0A0H3CQ97_ENTCC	0.017	+7.30

Table S4. Overlapping proteins identified between the LSMMG/NG and INV/NG comparisons. The table lists 26 proteins with consistent fold-change patterns across both comparisons.

Identified overlapping proteins LSMMG/NG and INV/NG	Accession Number	LSMMG/NG (FC)	INV/NG (FC)
Deoxyuridine 5'-triphosphate nucleotidohydrolase	A0A0H3CDN4_ENTCC	-12.50	-12.50
Mannitol-1-phosphate 5-dehydrogenase	A0A0H3CGQ1_ENTCC	-11.11	-11.11
Maltodextrin-binding protein	A0A0H3CJY1_ENTCC	-10.00	-50.00
Glutathione S-transferase domain-containing protein	A0A0H3CKK2_ENTCC	-10.00	-10.00
Core-binding (CB) domain-containing protein	A0A0H3CH80_ENTCC	-5.00	-5.00
D-isomer specific 2-hydroxyacid dehydrogenase, NAD-binding protein	A0A0H3CMB8_ENTCC	-5.00	-5.00
Triosephosphate isomerase	A0A0H3CTK6_ENTCC	-2.50	-2.50
3-ketoacyl-CoA thiolase FadA	A0A0H3CT88_ENTCC	-2.50	-2.50
Outer membrane protein W	A0A0H3CHT9_ENTCC	-2.00	-2.00
Co-chaperonin GroES	A0A0H3CG35_ENTCC	-1.67	-2.00
Chaperonin GroEL	A0A0H3CHT6_ENTCC	-1.67	-2.00
Chaperone protein DnaK	A0A0H3CGH6_ENTCC	-1.67	-2.00
Alkyl hydroperoxide reductase C	A0A0H3CHF3_ENTCC	-1.67	-2.00
AcrA protein	A0A0H3CGL3_ENTCC	-1.67	-1.67
Pyrroline-5-carboxylate reductase	A0A0H3CGD3_ENTCC	-1.67	-2.50
Transcriptional regulator HU subunit alpha	A0A0H3CDC6_ENTCC	-1.67	-2.00
Fumarate hydratase class I	A0A0H3CLM8_ENTCC	+1.70	+1.80
2,3-bisphosphoglycerate-independent phosphoglycerate mutase	A0A0H3CEN0_ENTCC	+1.80	+2.20
DNA topoisomerase 1	A0A0H3CJ11_ENTCC	+2.10	+2.60
Protein phosphatase CheZ	A0A0H3CIJ4_ENTCC	+2.30	+2.30
50S ribosomal protein L32	A0A0H3CKF0_ENTCC	+2.40	+1.70
Bactoprenol glucosyl transferase	A0A0H3CMZ4_ENTCC	+2.60	+2.00
Bifunctional polymyxin resistance protein ArnA	A0A0H3CSZ9_ENTCC	+2.70	+3.60
Putative fimbrial protein	A0A0H3CDJ5_ENTCC	+2.80	+2.10
Catalase-peroxidase KatG	A0A0H3CTH7_ENTCC	+3.10	+2.00
Sulfurtransferase	A0A0H3CNB4_ENTCC	+4.30	+2.80

Table S5. Overlapping proteins identified between the LSMMG/INV and LSMMG/NG comparisons. The table includes five proteins with shared fold-change directionality in both comparisons.

Identified overlapping proteins LSMMG/INV and LSMMG/NG	Accession Number	LSMMG/INV (FC)	LSMMG/NG (FC)
HTH-type transcriptional regulator IscR	A0A0H3CP88_ENTCC	-10.00	-10.00
Ribulose-phosphate 3-epimerase	A0A0H3CUE2_ENTCC	-3.33	-5.00
Cysteine desulfurase IscS	A0A0H3CNC4_ENTCC	+2.10	+2.00
Glycine cleavage system H protein	A0A0H3CQ70_ENTCC	+2.20	+2.00
RNA polymerase-associated protein RapA	A0A0H3CEW2_ENTCC	+7.00	+2.60

Table S6. Overlapping proteins identified between the LSMMG/INV and INV/NG comparisons. The table highlights 10 proteins showing inverse fold-change relationships between the two comparisons.

Identified overlapping proteins LSMMG/INV and INV/NG	Accession Number	LSMMG/INV (FC)	INV/NG (FC)
30S ribosomal protein S19	A0A0H3CQI0_ENTCC	-3.33	+2.20
Iron uptake system component EfeO	A0A0H3CLG5_ENTCC	-3.33	+6.70
YncE family protein	A0A0H3CJT0_ENTCC	-3.33	+6.70
DNA protection during starvation protein Dps	A0A0H3CKP0_ENTCC	-2.00	+1.50
Flagellin	A0A0H3CK20_ENTCC	-1.67	+1.80
Periplasmic serine endoprotease DegP-like	A0A0H3CF98_ENTCC	+1.50	-2.00
Succinate--CoA ligase [ADP-forming] subunit alpha	A0A0H3CPV5_ENTCC	+1.60	-2.00
Arginine transporter, periplasmic binding protein	A0A0H3CP70_ENTCC	+1.70	-2.50
Aldose 1-epimerase	A0A0H3CMI2_ENTCC	+2.50	-3.33
RNase adapter protein RapZ	A0A0H3CQ97_ENTCC	+7.30	-10.00

Table S7. ShinyGO functional enrichment results for LSMMG/NG, INV/NG, and LSMMG/INV comparisons. Functional analysis of differentially abundant proteins was conducted using *ShinyGO* (ver. 0.80) with the *E. cloacae* ATCC 13047 STRING db. A minimum of two proteins per pathway was required, with redundancies removed and an FDR cutoff of 0.05. The table lists all functional enrichments with FDR < 0.05.

Pathway	Fold Enrichment	Enrichment FDR
**LSMMG/NG (80 functional enrichments):**		
Carbon metabolism, and Fatty acid degradation	6.590	0.002
Glycolytic process, and pentose-phosphate shunt	17.547	0.002
Intracellular	1.783	0.002
Carbon metabolism, and monocarboxylic acid catabolic process	5.654	0.003
Glycolytic process, and pentose phosphate pathway	14.967	0.003
Glycolysis, and erythrose-4-phosphate dehydrogenase activity	23.947	0.003
Cytoplasm	1.796	0.003
Stress response, and Chaperonin Cpn60	43.617	0.003
Small molecule metabolic process	2.535	0.004
Ion binding	2.040	0.004
Cellular anatomical entity	1.276	0.004
Pentose phosphate pathway, and Glycolysis	11.834	0.004
Organic acid metabolic process	3.029	0.005
Carboxylic acid metabolic process	3.006	0.008
Cellular process	1.386	0.009
Peptidase complex, and stress response	25.443	0.011
Transferase	2.741	0.011
Oxoacid metabolic process	2.873	0.011
Mixed, incl. iron-sulfur cluster assembly, and thioredoxin-like superfamily	8.481	0.013
2Fe-2S	21.809	0.014
Glycolysis	21.809	0.014
Organophosphate metabolic process	3.622	0.014
Cellular metabolic process	1.626	0.014
Metal-binding	3.023	0.017
Stress response, and peptidase complex	17.960	0.019
Nucleoside phosphorylase domain	50.887	0.019
Nucleoside phosphorylase superfamily	50.887	0.019
Phosphorylase superfamily	50.887	0.019
Generation of precursor metabolites and energy	4.718	0.020
Metabolic process	1.487	0.020
Sulfurtransferase activity	16.962	0.020
Iron	5.501	0.020
Carbohydrate derivative metabolic process	3.018	0.022
Sodium transport	40.709	0.024

Glycolytic process	15.266	0.024
Nucleoside diphosphate phosphorylation	14.539	0.024
ATP generation from ADP	15.266	0.024
Purine nucleoside diphosphate metabolic process	14.539	0.024
Purine ribonucleoside diphosphate metabolic process	14.539	0.024
Ribonucleoside diphosphate metabolic process	14.539	0.024
ADP metabolic process	15.266	0.024
Nucleotide phosphorylation	14.539	0.024
Oxidation-reduction process	2.579	0.024
Pyruvate	13.878	0.026
Catalytic activity	1.480	0.028
Antioxidant activity, and glutathione s-transferase, c-terminal	12.722	0.029
Citrate cycle (TCA cycle), and lipid oxidation	7.982	0.029
Pyruvate kinase, and phosphoenolpyruvate carboxykinase activity	33.925	0.029
Mixed, incl. stress response, and clp atpase, c-terminal	12.722	0.029
Phosphate-containing compound metabolic process	2.615	0.029
Glutamine family amino acid biosynthetic process	12.722	0.029
Nucleoside diphosphate metabolic process	12.722	0.029
Transferase activity, transferring sulfur-containing groups	12.722	0.029
Ribonucleotide metabolic process	5.531	0.035
Mixed, incl. fatty acid degradation, and acetyl-coa metabolic process	11.308	0.036
Nucleotide metabolic process	4.393	0.036
Transferase activity, transferring pentosyl groups	11.308	0.036
Primary metabolic process	1.603	0.036
Cellular lipid metabolic process	3.789	0.036
Response to antibiotic	7.142	0.036
2 iron, 2 sulfur cluster binding	11.308	0.036
Nucleoside monophosphate metabolic process, and pyrimidine metabolism	5.301	0.036
Monocarboxylic acid metabolic process	3.769	0.036
Purine-containing compound metabolic process	5.301	0.036
Phosphorus metabolic process	2.482	0.036
Carbohydrate metabolic process	2.788	0.037
Nucleoside phosphate metabolic process	4.270	0.037
Ribose phosphate metabolic process	5.193	0.037
Mixed, incl. citrate cycle (tca cycle), and lipid oxidation	6.785	0.039
Chaperone, and protein catabolic process	10.528	0.039
Catabolic process	2.714	0.043
Rhodanese-like domain	22.616	0.044
Rhodanese Homology Domain	22.616	0.044
Organonitrogen compound metabolic process	1.908	0.045
Mixed, incl. antioxidant activity, and glutathione s-transferase, c-terminal	9.541	0.047

Lipid metabolic process	3.475	0.047
Pyruvate metabolic process	9.252	0.050
Organophosphate catabolic process	9.252	0.050
Rhodanese-like domain superfamily	20.355	0.050
Magnesium	3.914	0.050
**INV/NG (33 functional enrichments):**		
Antioxidant activity, and glutathione s-transferase, c-terminal	15.865	0.001
Stress response, and Chaperonin Cpn60	36.262	0.001
Cellular anatomical entity	1.264	0.001
Mixed, incl. antioxidant activity, and glutathione s-transferase, c-terminal	11.899	0.002
Carbon metabolism, and Fatty acid degradation	5.022	0.002
Oxidation-reduction process	2.721	0.002
Intracellular	1.606	0.002
Glycolysis, and erythrose-4-phosphate dehydrogenase activity	23.076	0.003
Peptidase complex, and stress response	21.153	0.003
Oxidoreductase	4.161	0.003
Carbon metabolism, and monocarboxylic acid catabolic process	4.309	0.005
Phosphoglycerate mutase activity, and phosphoglycerate kinase	31.729	0.008
Stress response, and peptidase complex	14.931	0.011
Channel activity	5.695	0.016
Passive transmembrane transporter activity	5.695	0.016
Mixed, incl. terd domain, and von willebrand factor type a domain	12.692	0.018
Response to antibiotic	6.680	0.018
Mixed, incl. citrate cycle (tca cycle), and fatty acid degradation	5.417	0.019
Porin	21.153	0.020
Mixed, incl. iron-sulfur cluster assembly, and thioredoxin-like superfamily	6.346	0.021
Cytoplasm	1.515	0.022
Mixed, incl. stress response, and clp atpase, c-terminal	10.576	0.026
Glucose metabolic process	10.576	0.026
Tricarboxylic acid cycle	10.576	0.026
ATP metabolic process	7.379	0.027
Reactive oxygen species metabolic process, and redoxin	15.865	0.036
Glycolytic process, and pentose-phosphate shunt	8.753	0.044
Chaperone, and protein catabolic process	8.753	0.044
Citrate cycle (TCA cycle), and lipid oxidation	6.221	0.047
Protein folding	6.221	0.047
Purine ribonucleotide metabolic process	4.945	0.048
Glycolysis	13.598	0.048
Aerobic respiration	8.188	0.049
**LSMMG/INV (5 functional enrichments):**		
Intracellular	1.887	0.029

Cytoplasm	1.924	0.029
Cellular anatomical entity	1.326	0.029
Alpha-amino acid catabolic process	15.680	0.029
Cellular amino acid catabolic process	13.485	0.032

Table S8. Unique proteins identified in the LSMMG/INV comparison. The table lists 17 proteins uniquely differentially abundant in the LSMMG/INV comparison.

Unique proteins LSMMG/INV	Accession Number	LSMMG/INV (FC)
Chemotaxis regulatory protein CheY	A0A0H3CI56_ENTCC	-3.33
Cytoplasmic chaperone TorD family protein	A0A0H3CLU7_ENTCC	-3.33
Putative oxidoreductase	A0A0H3CIC3_ENTCC	-2.50
1,4-dihydroxy-2-naphthoyl-CoA synthase	A0A0H3CPN2_ENTCC	-2.50
3-oxoacyl-[acyl-carrier-protein] synthase 3	A0A0H3CNF2_ENTCC	-2.00
Protein RecA	A0A0H3CSP0_ENTCC	-1.67
Fructose-bisphosphate aldolase	A0A0H3CMX1_ENTCC	-1.67
Bifunctional protein PutA	A0A0H3CLH0_ENTCC	+1.50
Glucose-specific PTS system component	A0A0H3CPZ1_ENTCC	+1.50
UspA domain-containing protein	A0A0H3CQ31_ENTCC	+1.70
D-amino acid dehydrogenase	A0A0H3CIV3_ENTCC	+1.70
Protein-export protein SecB	A0A0H3CD34_ENTCC	+1.80
Argininosuccinate synthase	A0A0H3CSA7_ENTCC	+1.80
Signal recognition particle protein	A0A0H3CSE5_ENTCC	+2.80
Methylmalonate-semialdehyde dehydrogenase	A0A0H3CN53_ENTCC	+4.20
LysM domain-containing protein	A0A0H3CKM3_ENTCC	+6.30
Regulator of nucleoside diphosphate kinase	A0A0H3CMR3_ENTCC	+7.00
